# The impact of achievement goal orientations on perceived stress: the moderating role of proactive personality

**DOI:** 10.3389/fpsyg.2026.1715353

**Published:** 2026-02-11

**Authors:** Haichao Wu, Zhongyi Xin, Jianhao Chen

**Affiliations:** 1 Faculty of Education, Shaanxi Normal University, Xi’an, China; 2 Faculty of Education Science, Shaanxi Xueqian Normal University, Xi’an, China

**Keywords:** achievement goal orientations, challenge stressors, college students, hindrance stressors, proactive personality

## Abstract

**Objective:**

This study aimed to examine the interactive effects of achievement goal orientations and proactive personality on perceived stress among college students, with a particular focus on challenge and hindrance stressors.

**Methods:**

A total of 648 college students participated in the study. Achievement goal orientations were specified as predictors, proactive personality was examined as a moderator, and perceived stress (including challenge stressors and hindrance stressors) served as the outcome variables. Age and gender were included as control variables. Moderation analyses were conducted to test the interactive effects.

**Results:**

Results showed that proactive personality significantly moderated the relationship between performance-approach goals and challenge stressors, such that a higher level of proactive personality attenuated the positive association between performance-approach goals and challenge stressors. No significant moderation effects were found for other types of achievement goal orientations on challenge stressors. In contrast, for hindrance stressors, proactive personality consistently weakened the positive associations between all types of achievement goal orientations and perceived stress.

**Conclusion:**

These findings highlight the important role of proactive personality in shaping how achievement goal orientations influence stress perceptions. The study advances theoretical understanding of the combined effects of motivational and personality factors on stress appraisal and offers practical implications for developing stress management interventions for college students.

## 1 Introduction

Stress among college students has long been a topic of sustained scholarly interest. Prior research has approached this issue from multiple perspectives, including the sources of stress and their structural characteristics (Gong et al., 2023; Zhang et al., 2024), differences in stress coping styles and strategies (Waterhouse and Samra, 2024; Wong and Shek, 2025), and the effects of stress on mental health and behavioral outcomes (Hu and Sun, 2023). Collectively, these studies have contributed to a systematic understanding of how stress emerges and operates among college students, while also providing important theoretical and practical insights for the development of targeted stress intervention programs in educational settings.

Despite differences in research perspectives and methodologies, a prevailing consensus in the literature is that stress generally exerts detrimental effects on college students’ mental health and well-being. In contrast, recent studies in organizational and occupational contexts have begun to recognize the potential positive functions of stress. For instance, the relationship between stress and creativity has been shown to be nonlinear, with certain levels or forms of stress facilitating creative performance (Baer and Oldham, 2006). Building on this view, scholars have proposed that stress varies not only in intensity but also in type as perceived by individuals, leading to the distinction between challenge stressors and hindrance stressors (Podsakoff et al., 2023; Pindek et al., 2024). Research adopting this framework has demonstrated the dual effects of stress: challenge stressors are more likely to foster positive motivation and adaptive behaviors, whereas hindrance stressors tend to evoke negative psychological experiences and undermine performance (Crawford et al., 2021). Accordingly, existing studies have primarily focused on the differential outcomes associated with these two types of stress, while the antecedents of stress perception have received comparatively limited attention.

Nevertheless, individuals often differ markedly in their cognitive and behavioral responses to the same stressors (LePine, 2022), suggesting that stress perception may be shaped by individual internal characteristics. In particular, whether achievement goal orientation and proactive personality contribute to variations in the perception of challenge and hindrance stress remains an open empirical question.

Against this background, the present study extends stress perception research from organizational settings to the population of college students and examines whether students with different achievement goal orientations and varying levels of proactive personality differ in their perceptions of academic stress. By focusing on challenge stress perception and hindrance stress perception within academic contexts, this study seeks to advance understanding of the individual antecedents underlying differentiated stress perceptions.

### 1.1 The relationship between achievement goal orientations and stress perception

Based on research conducted among organizational employees, Cavanaugh et al. (2000) distinguished stress into challenge stress and hindrance stress according to differences in stress sources. Challenge stress refers to stressors that individuals perceive as manageable and conducive to their career development, whereas hindrance stress refers to job demands that are viewed as obstacles to personal growth or that interfere with or constrain individuals’ ability to achieve their goals (Cavanaugh et al., 2000). Building on this classification, subsequent studies have extensively examined the effects of different types of stress on employees in organizational contexts; however, relatively little research has focused on stress perception among college students.

Although there are substantial differences between organizational workplaces and university settings, both contexts share a common feature: individuals strive for personal growth and development under conditions of limited resources and constraints. Building on this shared characteristic, the present study conceptualizes challenge stress among college students as stress that students perceive as manageable and beneficial to their academic achievement and skill development. Examples of challenge stressors in school include tight assignment deadlines, demanding examinations, and complex group projects, which, while requiring effort, can promote motivation, learning, and adaptive coping strategies. In contrast, hindrance stress is defined as stress perceived as obstructive to students’ progress and learning. Typical examples include ambiguous grading criteria, excessive administrative requirements, or conflicts with peers in academic teams, which impede progress without providing opportunities for growth. Importantly, the present study does not aim to classify specific stressors as inherently challenging or hindering. Rather, it focuses on students’ subjective perceptions of stress, namely, whether they construe stress as an opportunity and challenge or as an obstacle and barrier. By incorporating these concrete examples from school, this study provides a more nuanced understanding of how different types of stressors are perceived by students, setting the stage for examining individual differences in stress perception.

According to cognitive appraisal theory (Lazarus and Folkman, 1984; Folkman et al., 1986), stress perception depends on individuals’ subjective evaluations of situational demands. Primary appraisal is influenced by individual traits, among which achievement goal orientation plays a central role in shaping perceptions of task demands and environmental stressors (Urhahne and Wijnia, 2023; Wu et al., 2023). Goal orientation refers to individuals’ basic tendencies in selecting and pursuing achievement goals within achievement contexts, and it represents a motivational factor that influences students’ academic performance (Alasqah, 2022; Dweck and Leggett, 1988; Dweck, 1999; Liu et al., 2023). Achievement goal theory (Dweck and Leggett, 1988; Elliot and McGregor, 2001) distinguishes between Mastery-approach, Mastery-avoidance, Performance-approach, and Performance-avoidance orientations. These orientations are associated with distinct patterns of self-efficacy, emotional arousal, and cognitive strategies (Elliot and McGregor, 2001; Lee et al., 2024), leading to different perceptions of challenge stressors and hindrance stressors.

For example, student with a Mastery-approach orientation, who emphasize competence development and deep learning strategies, are more likely to interpret demanding tasks as opportunities for growth, thus perceiving higher levels of challenge stressors (Dweck and Leggett, 1988; Fong and Schallert, 2023). Those with a Performance-approach orientation, despite potential risks of distraction and resource depletion (Crouzevialle and Butera, 2017), may still perceive tasks as challenges due to their focus on outperforming others. In contrast, individuals with a Performance-avoidance orientation, characterized by fear of failure and low self-efficacy, are inclined to interpret tasks as threats, thereby reducing perceptions of challenge stressors (Elliot, 1999). The effects of Mastery-avoidance orientation remain less conclusive (Linnenbrink and Pintrich, 2000), as such goals may both increase negative stress experiences and motivate additional effort. Based on these arguments, the following hypotheses are proposed:

*H1a*: College students’ mastery-approach orientation is positively correlated with their perceived challenge stressors.

*H1b*: College students’ performance-approach orientation is positively correlated with their perceived challenge stressors.

*H1c*: College students’ performance-avoidance orientation is negatively correlated with their perceived challenge stressors.

*H1d*: College students’ mastery-avoidance orientation is negatively correlated with their perceived challenge stressors.

With regard to hindrance stressors, individuals with a Mastery-approach orientation, emphasizing effort and strategy, are less likely to perceive high hindrance stress. Conversely, those with a Mastery-avoidance orientation are more prone to anxiety in the face of obstacles (Linnenbrink and Pintrich, 2000; Morganson and Woods, 2022). Performance-oriented individuals, in general, tend to perceive more hindrance stressors: Performance-approach individuals may overreact to external barriers, whereas Performance-avoidance individuals are likely to adopt maladaptive coping strategies that exacerbate stress. Accordingly, the following hypotheses are proposed:

*H2a*: College students’ mastery-approach orientation is negatively correlated with their perceived hindrance stressors.

*H2b*: College students’ mastery-avoidance orientation is positively correlated with their perceived hindrance stressors.

*H2c*: College students’ performance-approach orientation is positively correlated with their perceived hindrance stressors.

*H2d*: College students’ performance-avoidance orientation is positively correlated with their perceived hindrance stressors.

### 1.2 The moderating role of proactive personality

The construct of proactive personality was introduced by Bateman and Crant (1993) and defined as a stable tendency to take initiative in influencing one’s environment. As a relatively stable dispositional trait, proactive personality reflects individuals’ tendencies to take initiative, identify opportunities, and effect change across a wide range of contexts (Bateman and Crant, 1993). Individuals with high proactive personality do not merely adapt to their environments; rather, they actively shape and modify environmental conditions to align with their personal goals and values (Din et al., 2023).

A growing body of empirical research has demonstrated the beneficial role of proactive personality in the stress and coping process (Kilic et al., 2024). Specifically, proactive individuals tend to adopt problem-focused coping strategies, mobilize available resources, and engage in proactive behaviors that buffer the negative effects of stressors (Mammadov et al., 2024). In their influential framework of proactive personality in the stress and coping process, Bolger and Zuckerman (1995) proposed that proactive personality plays a key role in shaping how individuals perceive, appraise, and respond to stressful situations. Subsequent studies have further supported this perspective, showing that proactive personality is associated with more adaptive stress perceptions and coping outcomes (Parker and Sprigg, 1999; Park and DeFrank, 2018; Nielsen et al., 2023).

Despite these advances, relatively few studies have examined the joint influence of proactive personality and achievement goal orientation on individuals’ perceptions of stress. Achievement goal orientation provides individuals with a motivational framework that guides how they interpret task demands and performance situations, whereas proactive personality reflects a dispositional tendency to actively engage with and shape these situations. From this perspective, it is reasonable to expect that the effects of achievement goal orientation on perceived stress may vary depending on individuals’ levels of proactive personality. In particular, among college students, whether the relationship between achievement goal orientation and stress perception differs across levels of proactive personality remains an open question that warrants further investigation.

According to stress reappraisal theory (Lazarus, 1991; Lazarus and Folkman, 1987), individuals’ stress experiences are not determined solely by objective demands but by their subjective cognitive appraisals of those demands. Individuals with high proactive personality are more likely to reinterpret potentially stressful situations as challenges rather than threats, view stressors as opportunities for learning and growth, actively seek social support, or flexibly adjust their goals to reduce perceived threats (Luo et al., 2023). At the same time, proactive individuals are also more inclined to expose themselves to stressors by voluntarily seeking challenging tasks and high-responsibility roles, which may increase their awareness of challenge-related demands.

By contrast, individuals with low proactive personality tend to adopt a more passive orientation toward their environment, relying more heavily on external cues and situational constraints. Such individuals may be less likely to actively reframe stressors or mobilize coping resources, making them more susceptible to perceiving stressors as hindrances that obstruct goal attainment. Taken together, these theoretical and empirical considerations suggest that proactive personality may function as an important moderating variable in the relationship between achievement goal orientation and perceived stress. Based on this reasoning, the following hypotheses are proposed:

*H3a*: Proactive personality moderates the relationship between achievement goal orientation and perceptions of challenge stressors.

*H3b*: Proactive personality moderates the relationship between achievement goal orientation and perceptions of hindrance stressors.

### 1.3 The present study

Although prior research has explored the role of individual traits in stress perception within the framework of cognitive appraisal theory, the influence of achievement goal orientation requires further investigation. Different goal orientations may shape perceptions of challenge stressors and hindrance stressors through mechanisms such as self-efficacy, emotional arousal, and cognitive strategies, but the exact patterns remain unclear. Moreover, personality traits—particularly Proactive personality—may play a crucial moderating role in these relationships, yet systematic empirical evidence remains scarce.

**Figure 1:**
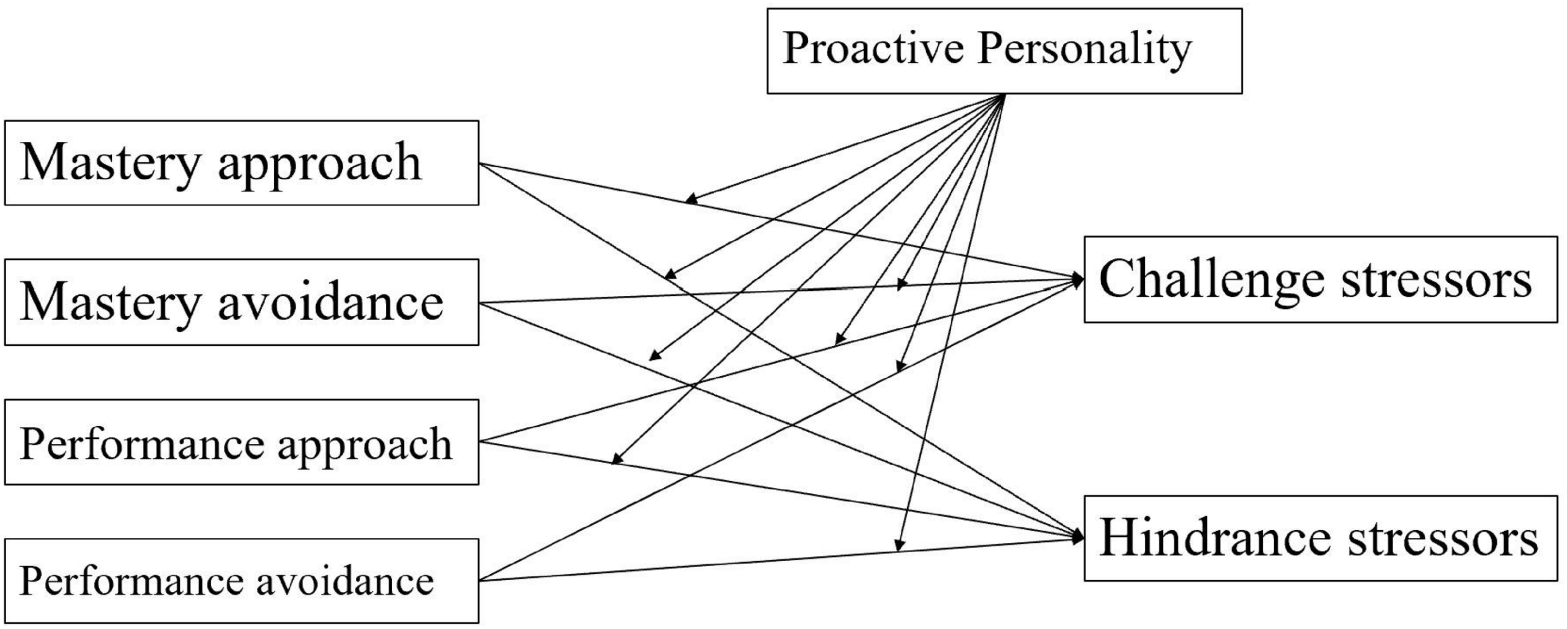
The proposed theoretical model.

To address these gaps, the present study proposed a research model (Figure 1) in which achievement goal orientation served as the independent variable, perceived stress—distinguished into challenge and hindrance stressors—served as the dependent variables, and proactive personality was specified as a moderator. Age and gender were included as control variables, as younger college students may experience higher levels of stress related to academic adjustment, whereas older students are more likely to encounter stress associated with career planning and employment transitions. Gender was also controlled due to potential differences in stress perception and stress responses. The study aimed to examine how stress perception varies across different achievement goal orientations and to explore the moderating role of proactive personality while controlling for age and gender. By doing so, potential confounding effects were minimized, allowing for a more accurate estimation of the relationships among achievement goal orientation, proactive personality, and perceived stress.

## 2 Participants and methods

### 2.1 Participants

A cluster sampling method was used to survey college students from two universities in Shaanxi Province. Student lists were provided by the student affairs offices, and questionnaires were distributed by research assistants and instructors after routine class meetings. Participants were informed of the study purpose, voluntary participation, anonymity, and absence of harm. Completing the questionnaire took approximately 5–10 min. After excluding responses with extremely short completion times or failed attention checks (e.g., “What is the capital of China?”), 648 valid questionnaires were retained, yielding a response rate of 92.8%. An *a priori* power analysis using G*Power 3.1 indicated that, for a small effect size (*f^2^* = 0.02), *α* = 0.05, power = 0.8, one tested predictor (interaction term), and three total predictors, the required sample size was approximately 395, indicating that the actual sample size of 648 was sufficient. Among participants, 167, 101, and 380 were in the first, second, and third years, respectively; 20.5% were male and 79.5% female, with a mean age of 19.2 years (SD = 1.15). Detailed information is presented in Table 1.

**Table 1:** Demographic characteristics of participants.

Variables category		*n*	%/(*M* ± SD)
Grade	First-year	167	25.8%
	Second-year	101	15.6%
	Third-year	380	58.6%
Gender	Male	133	20.5%
	female	515	79.5%
Age	/	648	19.2 ± 1.15

### 2.2 Measures

#### 2.2.1 Achievement goal orientation scale

The four-dimensional Achievement Goal Orientation Scale developed by Liu et al. (2006) was adopted. Based on the fourfold achievement goal framework, the scale distinguishes among mastery-approach, mastery-avoidance, performance-approach, and performance-avoidance goals, with a total of 29 items rated on a 5-point Likert scale (1 = strongly disagree to 5 = strongly agree). The mastery-approach subscale contains 9 items (e.g., “My greatest wish in class is to learn as much as possible.”), mastery-avoidance contains 5 items (e.g., “I always focus on the mistakes I make when studying”), performance-approach contains 9 items (e.g., “During learning, I am most afraid of being told by the teacher that I am not smart enough”), and performance-avoidance contains 6 items (e.g., “Before each exam, I always worry that there are topics I have not reviewed”). Dimension scores were calculated by averaging the respective items. In this study, the Cronbach’s *α* coefficient was 0.91 for the total scale, and 0.87, 0.87, 0.84, and 0.77 for the four dimensions, respectively.

#### 2.2.2 Challenge stressors and hindrance stressors scale

The Challenge Stressors and Hindrance Stressors Scale was originally developed by Cavanaugh et al. (2000) and later adapted into Chinese by Zhang and Lu (2009) for use with Chinese organizational employees. Since the present study focused on college students, whose primary responsibilities involve academic tasks, the Chinese version of the scale was further revised to reflect the stressors relevant to the school context. The scale comprises 11 items, including 6 items measuring challenge stressors (e.g., “I have considerable responsibilities related to my academic and student roles”) and 5 items measuring hindrance stressors (e.g., “I feel that my academic or personal development has stalled”). In this study, Cronbach’s *α* coefficients were 0.87 for challenge stressors and 0.76 for hindrance stressors.

#### 2.2.3 Proactive personality scale

The Proactive Personality Scale developed by Bateman and Crant (1993) and revised by Shang and Gan (2009) was used. It contains 11 items, rated on a 7-point Likert scale (1 = strongly disagree to 7 = strongly agree). A typical item is “I am constantly looking for better ways to do things.” Higher scores indicate higher levels of proactive personality. In this study, the Cronbach’s α coefficient was 0.84.

### 2.3 Data analysis

Statistical analyses were conducted using SPSS 25. Descriptive statistics and correlation analyses were first performed for all study variables. The four types of achievement goal orientation were treated as predictors, and challenge and hindrance stress served as outcome variables. Age and gender were included as control variables. Eight moderation models were constructed, and the PROCESS macro (version 4.0) was used to examine the moderating effects of proactive personality on the relationships between achievement goal orientations and both challenge and hindrance stress.

## 3 Results

### 3.1 Common method bias test

To examine potential common method bias, Harman’s single-factor test was conducted through exploratory factor analysis on all variables. The results showed that the first factor accounted for 21.77% of the variance, which did not exceed the critical threshold of 40%, indicating that common method bias was not a serious concern in this study and that the data demonstrated good reliability.

To examine the validity of the instruments used in this study, confirmatory factor analyses (CFA) were conducted for each questionnaire. The standardized factor loadings of the three questionnaires ranged from 0.419 to 0.895. The average variance extracted (AVE) values ranged from 0.321 to 0.536, and the composite reliability (CR) values ranged from 0.77 to 0.839, indicating acceptable convergent validity. In addition, the square roots of the AVE values were greater than the correlations between latent constructs, suggesting that discriminant validity was adequate (Table 2).

**Table 2:** Results of the discriminant validity assessment.

Variables	1	2	3	4	5	6	7
1. Mastery-approach	0.661						
2. Mastery-avoidance	0.657	0.66					
3. Performance-approach	0.374	0.051	0.723				
4. Performance-avoidance	0.621	0.624	0.522	0.635			
5. Challenge stressors	0.575	0.454	0.219	0.496	0.732		
6. Hindrance stressors	0.323	0.07	0.498	0.34	0.485	0.641	
7. Proactive personality	0.191	0.385	−0.092	0.248	0.212	−0.216	0.567

### 3.2 Descriptive statistics and correlation analysis

Table 3 presents the means, standard deviations, and correlation coefficients of the main variables. As shown in the table, mastery-approach, mastery-avoidance, and performance-approach were significantly positively correlated with challenge stressors (*r* = 0.50, *p* < 0.01; *r* = 0.40, *p* < 0.01; *r* = 0.20, *p* < 0.01). Performance-avoidance was also significantly positively correlated with challenge stressors (*r* = 0.41, *p* < 0.01). Mastery-approach was significantly positively correlated with hindrance stressors (*r* = 0.29, *p* < 0.01). In addition, mastery-avoidance, performance-approach, and performance-avoidance were significantly positively correlated with hindrance stressors (*r* = 0.11, *p* < 0.01; *r* = 0.47, *p* < 0.01; *r* = 0.25, *p* < 0.01, respectively). Finally, proactive personality showed significant positive correlations with both challenge stressors (*r* = 0.18, *p* < 0.01) and hindrance stressors (*r* = 0.17, *p* < 0.01).

**Table 3:** Means, standard deviations, and correlations among variables.

Variables	*M*	*SD*	1	2	3	4	5	6	7	8	9
1. Gender	–	–	1								
2. Age	21.93	1.41	0.07^*^	1							
3. Mastery-approach	3.37	0.56	−0.06	0.02	1						
4. Mastery-avoidance	3.44	0.52	0.02	0.09^*^	0.58^**^	1					
5. Performance-approach	2.93	0.68	0.02	0.02	0.33^**^	0.07	1				
6. Performance-avoidance	3.47	0.55	0.03	−0.01	0.59^**^	0.51^**^	0.41^**^	1			
7. Challenge stressors	19.90	3.17	0.01	0.14^**^	0.50^**^	0.40^**^	0.20^**^	0.41^**^	1		
8. Hindrance stressors	15.18	2.68	−0.05	0.07	0.29^**^	0.11^**^	0.47^**^	0.25^**^	0.45^**^	1	
9. Proactive personality	53.26	9.07	0.15^**^	0.13^**^	0.17^**^	0.33^**^	−0.10^*^	0.20^**^	0.18^**^	0.17^**^	1

*p <0.05; **p <0.05.

### 3.3 Testing the moderating effect of proactive personality

To test the proposed hypotheses, eight separate moderation models were constructed. Table 4 presents the results for H3a, and Table 5 presents the results for H3b. Models 1 to 4 examined the effects of achievement goal orientation on challenge stressors and the moderating role of Proactive personality, while Models 5 to 8 examined the effects on hindrance stressors.

**Table 4:** Moderating effects of proactive personality on the relationship between achievement goal orientation and challenge stressors.

Outcome	Predictors	*β*	SE	*t*	*p*	LLCI	ULCI	*R^2^*	*∆R^2^*	*F*
Model 1: challenge stressors	Gender	−0.002	0.085	−0.020	0.984	−0.168	0.165	0.274	0.0003	48.344
	Age	0.085	0.024	3.501	<0.001	0.037	0.133			
	Mastery approach	0.483	0.035	13.911	<0.001	0.415	0.551			
	Proactive personality	0.079	0.035	2.273	0.023	0.011	0.148			
	Mastery approach*Proactive personality	0.015	0.033	0.471	0.638	−0.049	0.079			
Model 2: challenge stressors	Gender	−0.048	0.090	−0.536	0.592	−0.226	0.129	0.172	0.009	26.711
	Age	0.066	0.026	2.543	0.011	0.015	0.118			
	Mastery avoidance	0.378	0.038	9.889	<0.001	0.303	0.453			
	Proactive personality	0.045	0.039	1.169	0.243	−0.031	0.121			
	Mastery avoidance*Proactive personality	−0.030	0.036	−0.850	0.396	−0.100	0.040			
Model 3: challenge stressors	Gender	−0.095	0.093	−1.018	0.309	−0.278	0.088	0.112	0.0204	16.172
	Age	0.076	0.027	2.832	0.005	0.023	0.128			
	Performance approach	0.254	0.039	6.542	<0.001	0.178	0.330			
	Proactive personality	0.195	0.038	5.131	<0.001	0.121	0.270			
	Performance approach*Proactive personality	−0.155	0.040	−3.841	<0.001	−0.234	−0.076			
Model 4: Challenge stressors	Gender	−0.099	0.089	−1.116	0.265	−0.273	0.075	0.196	0.0001	31.336
	Age	0.094	0.026	3.683	<0.001	0.044	0.145			
	Performance avoidance	0.396	0.036	10.910	<0.001	0.325	0.467			
	Proactive personality	0.089	0.037	2.412	0.016	0.017	0.161			
	Performance avoidance*Proactive personality	−0.006	0.036	−0.162	0.871	−0.076	0.064			

**Table 5:** Moderating effects of proactive personality on the relationship between achievement goal orientation and hindrance stressors.

Outcome	Predictors	*β*	SE	*t*	*p*	LLCI	ULCI	*R^2^*	*∆R2*	*F*
Model 5: Hindrance stressors	Gender	0.044	0.090	0.489	0.625	−0.133	0.221	0.178	0.029	22.788
	Age	0.060	0.026	2.317	0.021	0.009	0.110			
	Mastery approach	0.362	0.037	9.803	<0.001	0.290	0.435			
	Proactive personality	−0.245	0.037	−6.602	<0.001	−0.317	−0.172			
	Mastery approach*Proactive personality	−0.165	0.035	−4.778	<0.001	−0.233	−0.097			
Model 6: Hindrance stressors	Gender	−0.002	0.093	−0.024	0.981	−0.186	0.181	0.115	0.044	16.741
	Age	0.042	0.027	1.546	0.123	−0.011	0.095			
	Mastery avoidance	0.204	0.040	5.165	<0.001	0.127	0.282			
	Proactive personality	−0.253	0.040	−6.339	<0.001	−0.332	−0.175			
	Mastery avoidance*Proactive personality	−0.209	0.037	−5.670	<0.001	−0.281	−0.136			
Model 7: Hindrance stressors	Gender	−0.083	0.086	−0.967	0.334	−0.251	0.085	0.255	0.006	43.988
	Age	0.059	0.025	2.420	0.016	0.011	0.107			
	Performance approach	0.480	0.036	13.501	0.000	0.410	0.549			
	Proactive personality	−0.137	0.035	−3.917	0.000	−0.205	−0.068			
	Performance approach*Proactive personality	−0.082	0.037	−2.229	0.026	−0.155	−0.010			
Model 8: Hindrance stressors	Gender	−0.051	0.092	−0.557	0.578	−0.232	0.129	0.136	0.009	20.157
	Age	0.072	0.027	2.707	0.007	0.020	0.124			
	Performance avoidance	0.308	0.038	8.183	0.000	0.234	0.382			
	Proactive personality	−0.252	0.038	−6.588	0.000	−0.327	−0.177			
	Performance avoidance*Proactive personality	−0.095	0.037	−2.559	0.011	−0.168	−0.022			

To examine whether proactive personality moderates the relationship between mastery-approach goal orientation and perceived challenge stressors, Model 1 was constructed by including gender, age, mastery-approach goal orientation, proactive personality, and their interaction term. Results indicated that the overall model was significant (*R*^2^ = 0.274, Δ*R*^2^ = 0.0003, *F* = 48.344). Specifically, after controlling for gender and age, mastery-approach goal orientation significantly and positively predicted challenge stressors (*β* = 0.483, SE = 0.035, *t* = 13.911, *p* < 0.001, 95% CI [0.415, 0.551]), and proactive personality also showed a positive predictive effect (*β* = 0.079, SE = 0.035, *t* = 2.273, *p* = 0.023, 95% CI [0.011, 0.148]). However, the interaction term was not significant (*β* = 0.015, SE = 0.033, *t* = 0.471, *p* = 0.638, 95% CI [−0.049, 0.079]), suggesting that proactive personality did not significantly moderate the association between mastery-approach goal orientation and challenge stressors. The increase in explained variance after adding the interaction term was minimal (Δ*R*^2^ = 0.0003).

To test whether proactive personality moderates the relationship between mastery-avoidance goal orientation and perceived challenge stressors, Model 2 was constructed by entering gender, age, mastery-avoidance goal orientation, proactive personality, and their interaction term. Results showed that the overall model was significant (*R*^2^ = 0.172, Δ*R*^2^ = 0.009, *F* = 26.711). After controlling for gender and age, mastery-avoidance goal orientation positively predicted challenge stressors (*β* = 0.378, SE = 0.038, *t* = 9.889, *p* < 0.001, 95% CI [0.303, 0.453]), and age also exhibited a significant positive effect (*β* = 0.066, SE = 0.026, *t* = 2.543, *p* = 0.011, 95% CI [0.015, 0.118]). However, the interaction between mastery-avoidance goal orientation and proactive personality was not significant (*β* = −0.030, SE = 0.036, *t* = −0.850, *p* = 0.396, 95% CI [−0.100, 0.040]), indicating that proactive personality does not significantly moderate the effect of mastery-avoidance goal orientation on challenge stressors. Although adding the interaction term slightly increased the explained variance, the increment was small (Δ*R*^2^ = 0.009).

**Figure 2:**
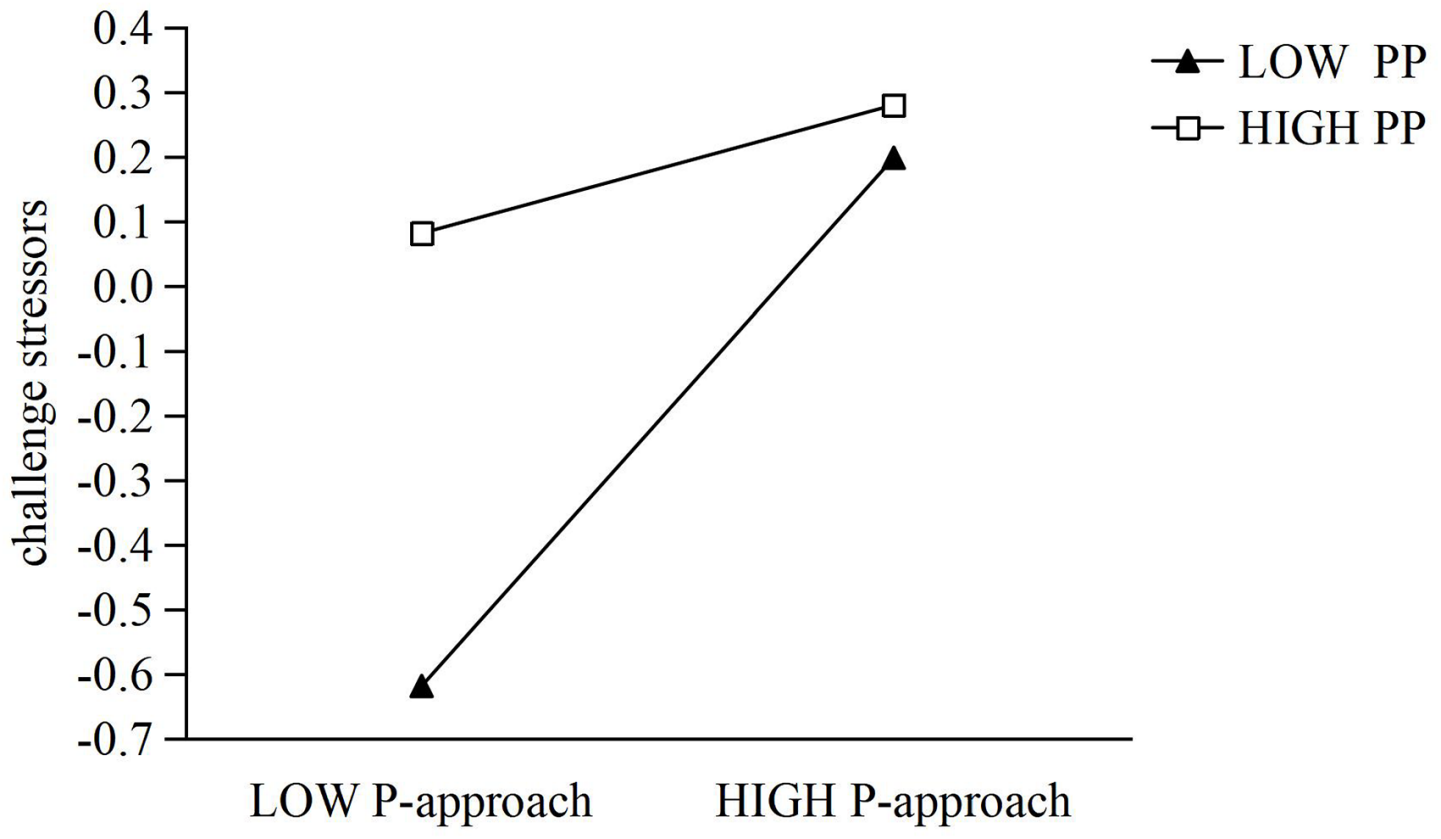
The moderating effect of proactive personality on the relationship between performance-approach and challenge stressors.

To investigate whether proactive personality moderates the relationship between performance-approach goal orientation and perceived challenge stressors, Model 3 was constructed by including gender, age, performance-approach goal orientation, proactive personality, and their interaction term. The results demonstrated that the overall model was significant (*R*^2^ = 0.112, Δ*R*^2^ = 0.0204, *F* = 16.172). After controlling for gender and age, performance-approach goal orientation significantly and positively predicted challenge stressors (*β* = 0.254, SE = 0.039, *t* = 6.542, *p* < 0.001, 95% CI [0.178, 0.330]), and proactive personality also showed a significant positive effect (*β* = 0.195, SE = 0.038, *t* = 5.131, *p* < 0.001, 95% CI [0.121, 0.270]). Notably, the interaction term reached significance (*β* = −0.155, SE = 0.040, *t* = −3.841, *p* < 0.001, 95% CI [−0.234, −0.076]), indicating that proactive personality significantly moderated the effect of performance-approach goal orientation on challenge stressors. To further clarify the moderating role of proactive personality, simple slope analyses were conducted by examining the effect of performance-approach goal orientation on challenge stressors at high and low levels of proactive personality (i.e., ±1 SD from the mean). The results showed that when proactive personality was high, performance-approach goal orientation significantly and positively predicted challenge stressors (*b* = 0.093, SE = 0.048, *t* = 2.074, 95% CI [0.005, 0.193]). As illustrated in Figure 2, when proactive personality was low, performance-approach goal orientation also significantly and positively predicted challenge stressors (*b* = 0.408, SE = 0.063, *t* = 6.492, 95% CI [0.285, 0.532]). These findings indicate that the positive effect of performance-approach goal orientation on challenge stressors is weaker among individuals with higher proactive personality and stronger among those with lower proactive personality.

To examine whether proactive personality moderates the relationship between performance-avoidance goal orientation and perceived challenge stressors, Model 4 was constructed by entering gender, age, performance-avoidance goal orientation, proactive personality, and their interaction term. Results indicated that the overall model was significant (*R*^2^ = 0.196, Δ*R*^2^ = 0.0001, *F* = 31.336). After controlling for gender and age, performance-avoidance goal orientation positively predicted challenge stressors (*β* = 0.396, SE = 0.036, *t* = 10.910, *p* < 0.001, 95% CI [0.325, 0.467]), and proactive personality also showed a significant positive effect (*β* = 0.089, SE = 0.037, *t* = 2.412, *p* = 0.016, 95% CI [0.017, 0.161]). However, the interaction term was not significant (*β* = −0.006, SE = 0.036, *t* = −0.162, *p* = 0.871, 95% CI [−0.076, 0.064]), suggesting that proactive personality does not moderate the relationship between performance-avoidance goal orientation and challenge stressors. Additionally, the contribution of the interaction term to the explained variance was negligible (Δ*R*^2^ = 0.0001).

**Figure 3:**
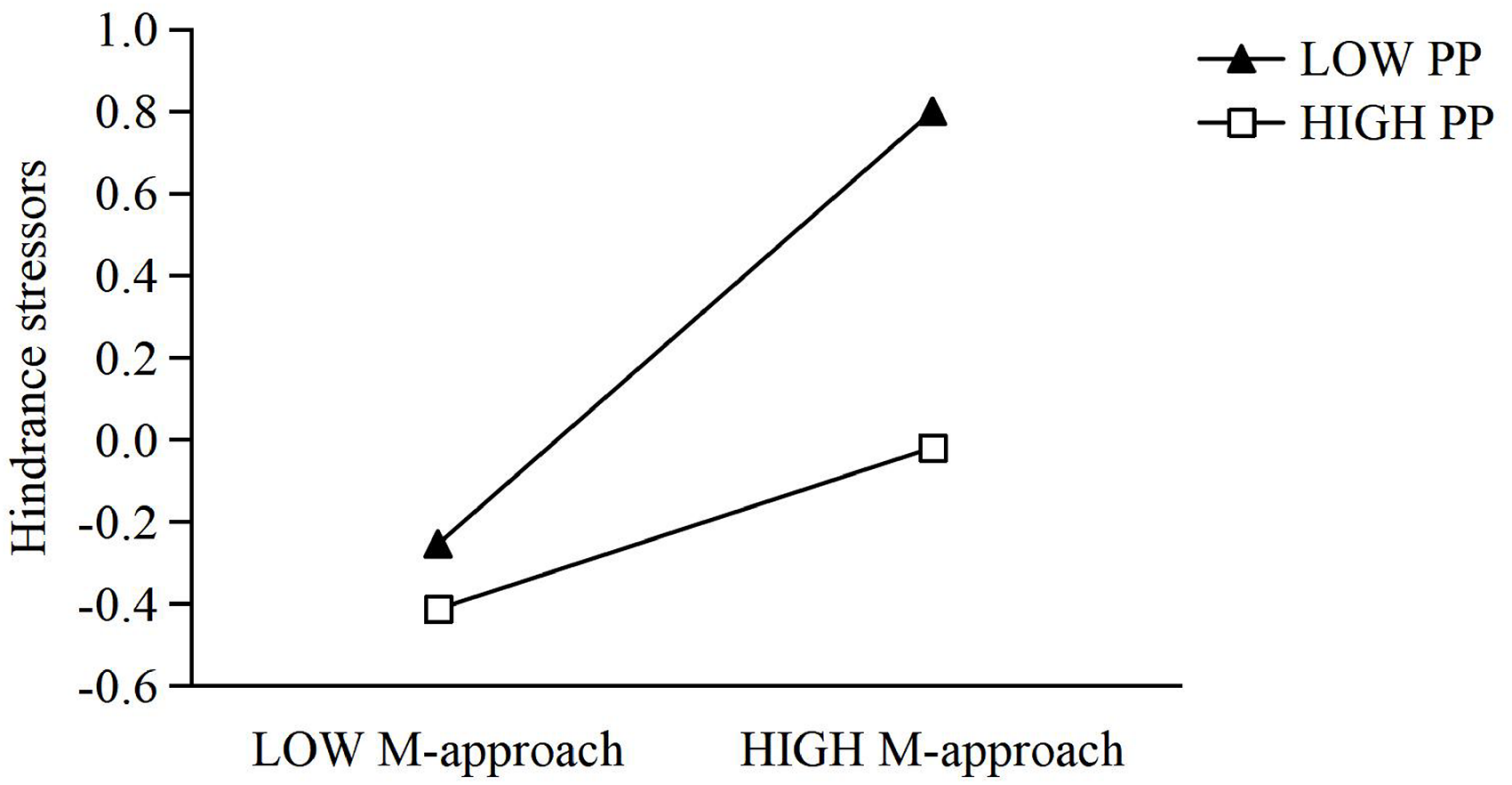
The moderating effect of proactive personality on the relationship between mastery-approach and hindrance stressors.

To examine whether proactive personality moderates the relationship between mastery-approach goal orientation and hindrance stressors, as shown in Table 5, Model 5 was constructed by including gender, age, mastery-approach goal orientation, proactive personality, and their interaction term. The overall model was significant (*R*^2^ = 0.178, Δ*R*^2^ = 0.029, *F* = 22.788). After controlling for gender and age, mastery-approach goal orientation significantly predicted hindrance stressors (*β* = 0.362, SE = 0.037, *t* = 9.803, *p* < 0.001, 95% CI [0.290, 0.435]), whereas proactive personality negatively predicted them (*β* = −0.245, SE = 0.037, *t* = −6.602, *p* < 0.001, 95% CI [−0.317, −0.172]). The interaction term was also significant (*β* = −0.165, SE = 0.035, *t* = −4.778, *p* < 0.001, 95% CI [−0.233, −0.097]), indicating that proactive personality moderated the association between mastery-approach orientation and hindrance stressors. To further clarify the moderating effect of proactive personality, a simple slopes analysis was conducted using values of proactive personality at one standard deviation below the mean, at the mean, and one standard deviation above the mean. The results showed that when proactive personality was low (−1 SD), mastery-approach goal orientation strongly and positively predicted hindrance stressors (*b* = 0.5273, SE = 0.0545, *t* = 9.676, *p* < 0.001, 95% CI [0.4203, 0.6343]). At high levels of proactive personality (+1 SD), the predictive effect of mastery-approach goal orientation on hindrance stressors remained significant but was weaker (*b* = 0.1968, SE = 0.0464, *t* = 4.243, *p* < 0.001, 95% CI [0.1057, 0.2878]). These results indicate that higher levels of proactive personality attenuate the positive association between mastery-approach goal orientation and hindrance stressors. The detailed moderating effect is illustrated in Figure 3.

**Figure 4:**
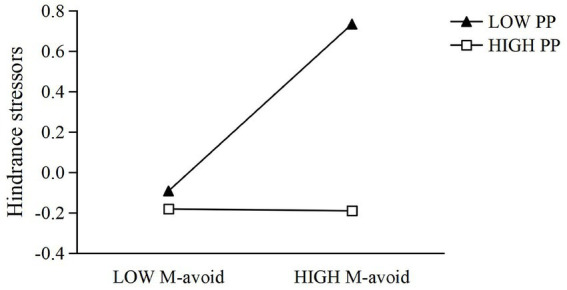
The moderating effect of proactive personality on the relationship between mastery-avoidance and hindrance stressors.

To further test the moderating role of proactive personality, Model 6 incorporated gender, age, mastery-avoidance goal orientation, proactive personality, and their interaction term. The model was significant (*R*^2^ = 0.115, Δ*R*^2^ = 0.044, *F* = 16.741). Controlling for gender and age, mastery-avoidance goal orientation significantly predicted hindrance stressors (*β* = 0.204, SE = 0.040, *t* = 5.165, *p* < 0.001, 95% CI [0.127, 0.282]), and proactive personality had a significant negative effect (*β* = −0.253, SE = 0.040, *t* = −6.339, *p* < 0.001, 95% CI [−0.332, −0.175]). The interaction term was significant (*β* = −0.209, SE = 0.037, *t* = −5.670, *p* < 0.001, 95% CI [−0.281, −0.136]), indicating a significant moderating effect of proactive personality on the relationship between mastery-avoidance orientation and hindrance stressors. To further clarify the moderating effect of proactive personality, a simple slopes analysis was conducted using values of proactive personality at one standard deviation below the mean, at the mean, and one standard deviation above the mean. The results showed that when proactive personality was low (−1 SD), mastery-avoidance goal orientation positively predicted hindrance stressors (*b* = 0.4128, SE = 0.0565, *t* = 7.302, *p* < 0.001, 95% CI [0.3018, 0.5238]). At the mean level of proactive personality, the effect remained significant but was weaker (*b* = 0.2042, SE = 0.0395, *t* = 5.165, *p* < 0.001, 95% CI [0.1266, 0.2819]). At high levels of proactive personality (+1 SD), the effect of mastery-avoidance goal orientation on hindrance stressors was no longer significant (*b* = −0.0043, SE = 0.0514, *t* = −0.084, *p* = 0.932, 95% CI [−0.1052, 0.0965]). These results indicate that higher levels of proactive personality weaken and even neutralize the positive association between mastery-avoidance goal orientation and hindrance stressors. The detailed moderating effect is illustrated in Figure 4.

**Figure 5:**
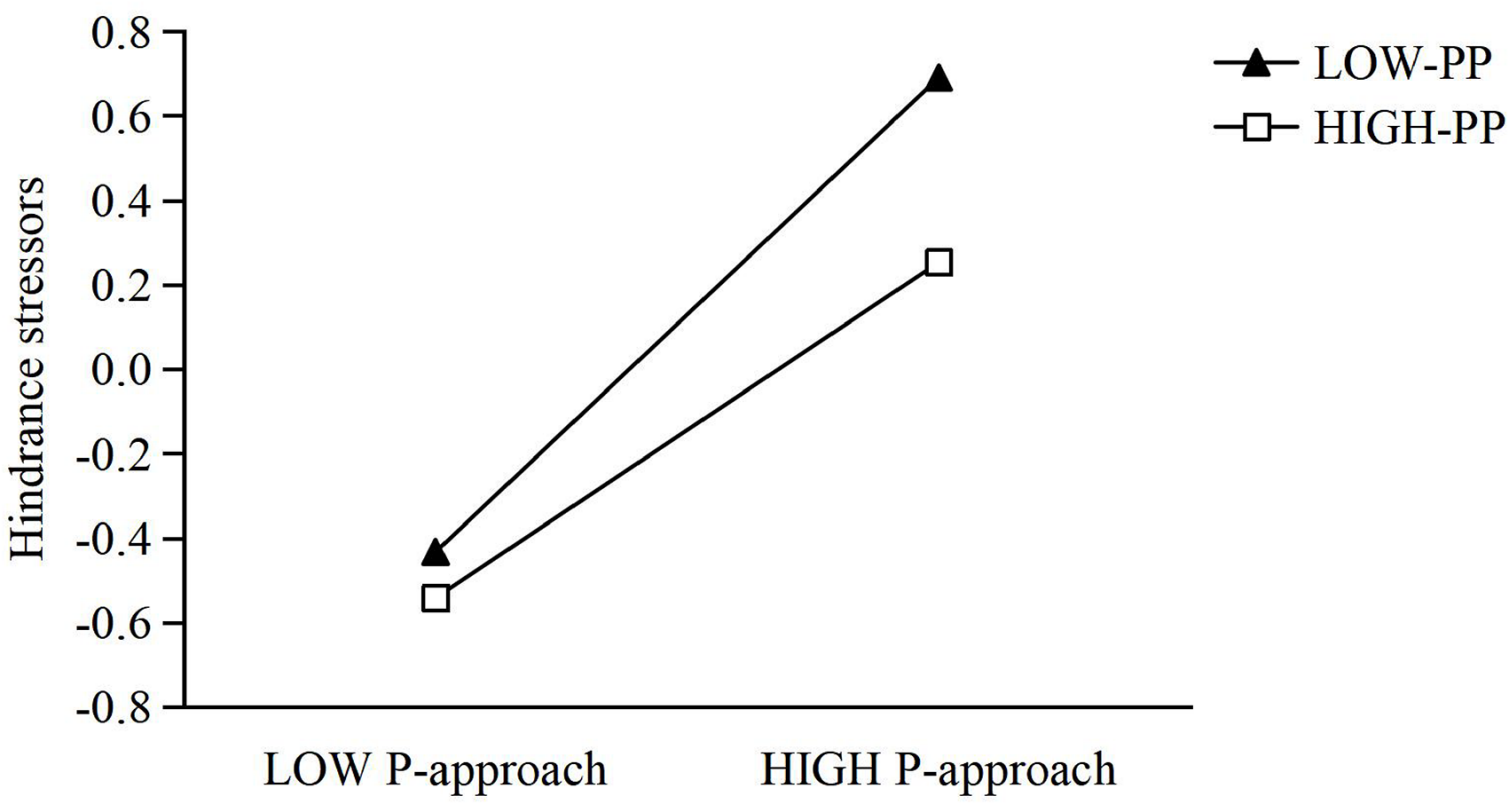
The moderating effect of proactive personality on the relationship between performance-approach and hindrance stressors.

To investigate whether proactive personality moderates the effect of performance-approach goal orientation on hindrance stressors, Model 7 included gender, age, performance-approach goal orientation, proactive personality, and their interaction term. The model was significant (*R*^2^ = 0.255, Δ*R*^2^ = 0.006, *F* = 43.988). After controlling for gender and age, performance-approach orientation significantly predicted hindrance stressors (*β* = 0.480, SE = 0.036, *t* = 13.501, *p* < 0.001, 95% CI [0.410, 0.549]), while proactive personality negatively predicted them (*β* = −0.137, SE = 0.035, *t* = −3.917, *p* < 0.001, 95% CI [−0.205, −0.068]). The interaction term reached significance (*β* = −0.082, SE = 0.037, *t* = −2.229, *p* = 0.026, 95% CI [−0.155, −0.010]), suggesting that proactive personality moderated the link between performance-approach orientation and hindrance stressors. To further clarify the moderating effect of proactive personality, a simple slopes analysis was conducted using values of proactive personality at one standard deviation below the mean, at the mean, and one standard deviation above the mean. The results showed that when proactive personality was low (−1 SD), performance-approach goal orientation strongly and positively predicted hindrance stressors (*b* = 0.5618, SE = 0.0576, *t* = 9.755, *p* < 0.001, 95% CI [0.4487, 0.6749]). At the mean level of proactive personality, the effect remained significant (*b* = 0.4797, SE = 0.0355, *t* = 13.501, *p* < 0.001, 95% CI [0.4099, 0.5494]). At high levels of proactive personality (+1 SD), the predictive effect, although still significant, was weaker (*b* = 0.3975, SE = 0.0438, *t* = 9.068, *p* < 0.001, 95% CI [0.3114, 0.4836]). These findings suggest that higher levels of proactive personality attenuate the positive association between performance-approach goal orientation and hindrance stressors. The detailed moderating effect is illustrated in Figure 5.

**Figure 6:**
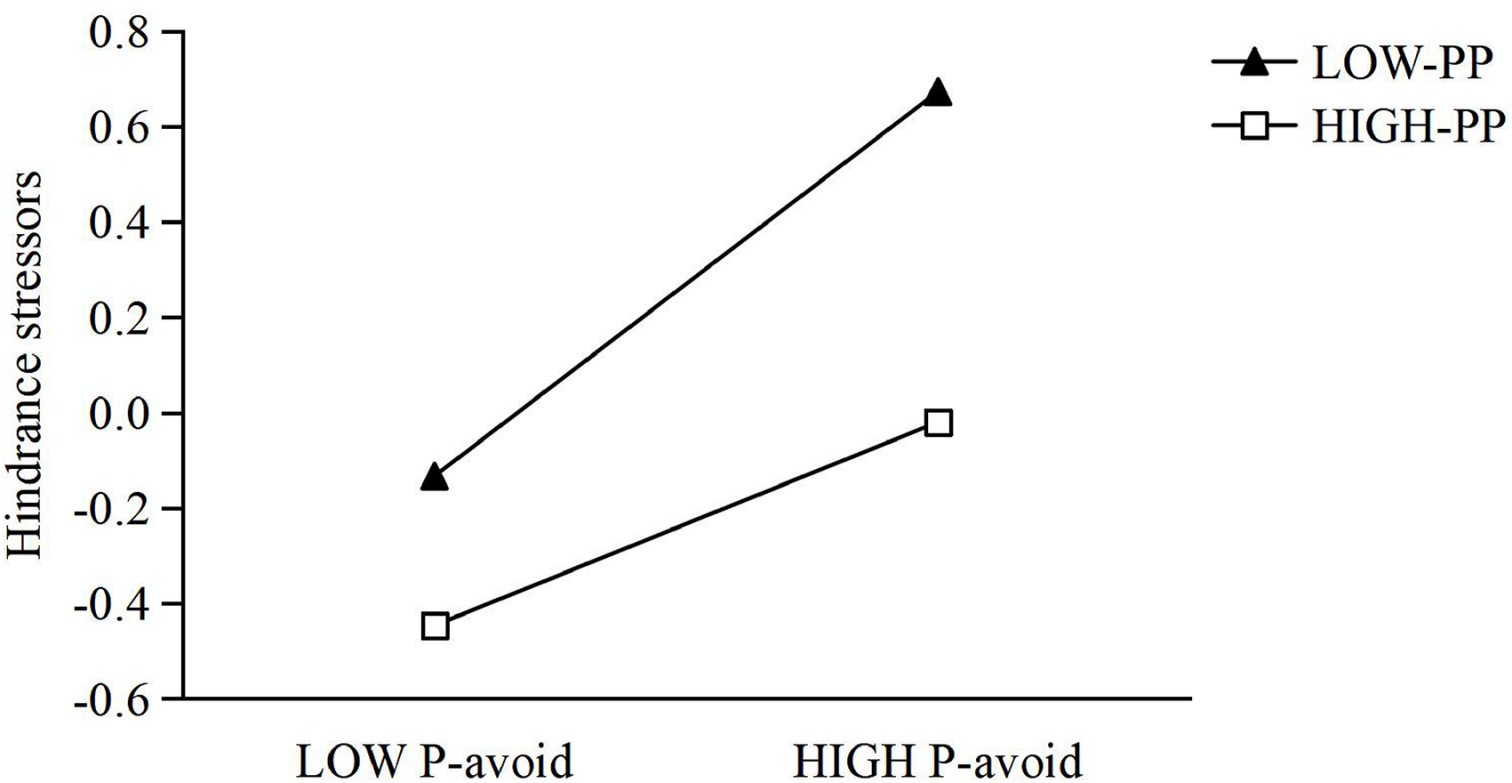
The moderating effect of proactive personality on the relationship between performance-avoidance and hindrance stressors.

To examine the moderating influence of proactive personality on the association between performance-avoidance goal orientation and hindrance stressors, Model 8 was constructed by entering gender, age, performance-avoidance goal orientation, proactive personality, and their interaction term. The model was significant (*R*^2^ = 0.136, Δ*R*^2^ = 0.009, *F* = 20.157). Controlling for gender and age, performance-avoidance goal orientation significantly predicted hindrance stressors (*β* = 0.308, SE = 0.038, *t* = 8.183, *p* < 0.001, 95% CI [0.234, 0.382]), and proactive personality again demonstrated a significant negative effect (*β* = −0.252, SE = 0.038, *t* = −6.588, *p* < 0.001, 95% CI [−0.327, −0.177]). The interaction term was significant (*β* = −0.095, SE = 0.037, *t* = −2.559, *p* = 0.011, 95% CI [−0.168, −0.022]), indicating that proactive personality moderated the relationship between performance-avoidance orientation and hindrance stressors. To further probe the interaction between performance-avoidance goal orientation and proactive personality, simple slope analyses were conducted at low (−1 SD), mean, and high (+1 SD) levels of proactive personality. The results indicated that performance-avoidance orientation was positively associated with hindrance stressors at low (*β* = 0.403, SE = 0.055, *t* = 7.276, *p* < 0.001, 95% CI [0.294, 0.512]), mean (*β* = 0.308, SE = 0.038, *t* = 8.183, *p* < 0.001, 95% CI [0.234, 0.382]), and high (*β* = 0.213, SE = 0.050, *t* = 4.247, *p* < 0.001, 95% CI [0.115, 0.312]) levels of proactive personality. Notably, the strength of this relationship decreased as proactive personality increased, suggesting that proactive personality buffered the positive effect of performance-avoidance orientation on hindrance stressors. The detailed moderating effect is illustrated in Figure 6.

## 4 Discussion

### 4.1 The moderating role of proactive personality in the relationship between achievement goal orientations and perceived stress

This study provides important theoretical insights into how different types of achievement goal orientation influence individuals’ perceptions of Challenge stressors and Hindrance stressors, as well as the boundary conditions and underlying mechanisms of these effects.

First, this study examined the impact of achievement goal orientation on perceived stress. Previous research has suggested that Mastery-approach goals tend to buffer Hindrance stressors, whereas Performance-avoidance goals may suppress Challenge stressors (Elliot and McGregor, 2001). However, our analyses across eight different models revealed that Mastery-approach, Mastery-avoidance, Performance-approach, and Performance-avoidance all positively predicted both Challenge stressors and Hindrance stressors. This finding diverges from previous research (Ma et al., 2021), likely because the participants in this study were university students facing dual pressures from academic competition and career transitions. In such a context, even goals traditionally considered “adaptive” may become stressors due to limited learning resources or excessively high self-demands. Moreover, Performance-avoidance goals, in the absence of structured support in an open learning environment, may lead individuals to perceive challenging tasks as uncontrollable threats, thus exhibiting a positive association with Challenge stressors (Lee and Kim, 2014).

These results suggest that the nature of Challenge stressors and Hindrance stressors is not fixed but depends on the fit between individual goals and environmental conditions. For instance, a Mastery-approach student facing time pressure may experience frustration in meeting growth needs, transforming potential challenges into Hindrance stressors. Similarly, a Performance-avoidance student in a high-stakes assessment context may reinterpret otherwise challenging tasks as obstacles due to self-doubt. Therefore, the effect of achievement goal orientation on stress perception is context-dependent. This study not only addresses gaps in previous research but also provides new evidence for the development of achievement goal theory, particularly by enriching our understanding of the mechanisms of Mastery-approach and Performance-avoidance goals from an interactionist perspective (Ames, 1992). It highlights that traditionally “adaptive” goals may lose their protective role during transitional periods, such as university.

Second, this study examined the moderating role of Proactive personality in the relationship between achievement goal orientation and perceived stress, revealing important boundary conditions. For challenge stressors, proactive personality showed a significant moderating effect only for performance-approach goals, but not for mastery-approach, mastery-avoidance, or performance-avoidance goals. This suggests differential mechanisms in the stress transformation process across goal types. The core of Performance-approach goals is to surpass others; this competitive tendency, combined with the action-oriented and initiative-driven traits of Proactive personality, enables individuals to convert stress into motivation effectively. High Proactive personality can attenuate excessive stress associated with performance-approach goals, making stress more controllable and constructive. In contrast, mastery goals already emphasize deep engagement and resource investment, overlapping with the mechanisms of proactive personality, so additional moderating effects are not observed. These findings align with achievement goal theory regarding the link between Performance-approach goals and challenge appraisal (Nicholls et al., 2014), indicating that proactive personality does not universally amplify all goal types but is particularly salient under Performance-approach conditions.

Finally, this study confirmed the general moderating effect of Proactive personality on the relationship between achievement goal orientation and Hindrance stressors, supporting H3b. Specifically, Proactive personality attenuated the positive association between all types of achievement goals and Hindrance stressors, consistent with cognitive appraisal theory (Lazarus and Folkman, 1984), which posits that individual traits can reshape the psychological meaning of external stimuli. High Proactive personality facilitates more adaptive interpretations of goal-related stress, reducing its perceived threat. Notably, the buffering effect is not uniform; under low Proactive personality, the positive effect of achievement goals on Hindrance stressors is stronger, whereas high Proactive personality significantly mitigates this effect. This aligns with prior research (Wei et al., 2021) showing that proactive individuals can buffer adverse stress perceptions through positive cognition and resource mobilization. It also resonates with Parker et al.’s (2010) concept of the “adaptive boundary” of proactive behaviors, highlighting the need for sufficient resources and realistic goal setting to support proactive functioning. Without adequate resources, even highly proactive individuals may experience elevated Hindrance stressors due to goal–resource mismatch. Therefore, fostering Proactive personality in educational or organizational settings should be coupled with environmental support and realistic goal alignment to prevent additional stress.

### 4.2 Practical implications

The findings provide practical implications for stress management among university students. First, educators should avoid simplistic classifications of achievement goal orientations. Even traditionally adaptive mastery-approach goals can contribute to hindrance stressors under high task demands or limited resources; guiding students to dynamically align goals with situational conditions is recommended. Second, environmental support is particularly important for students with performance-avoidance goals, who are prone to elevated hindrance stressors in competitive or high-evaluation contexts. Structured guidance, phased feedback, and diverse assessment strategies can enhance perceived control and reduce stress accumulation. Third, proactive personality should be guided and managed effectively. While proactive students tend to interpret stress as manageable challenges and buffer the impact of achievement goals on hindrance stressors, overcommitment without adequate resources may lead to resource depletion and increased stress. Educators should assist students in realistic goal-setting, responsibility management, and provision of supportive resources. Overall, effective stress interventions should integrate goal adjustment, environmental support, and trait management to maximize constructive stress and minimize obstructive stress.

### 4.3 Limitations

Despite its valuable findings, this study has several limitations. First, regarding the research design, the cross-sectional approach restricts the ability to draw causal inferences between achievement goal orientation and perceived stress. Future studies could employ longitudinal or experimental designs to better identify causal pathways and potential lagged effects, thereby improving the robustness and generalizability of the findings.

Second, there are measurement limitations. The scales used to assess challenge and hindrance stressors primarily capture short-term stress states and do not differentiate between short-term and long-term stress experiences. Future research should incorporate longitudinal data or multi-level indicators to examine stress perceptions across different temporal dimensions.

Third, the present study relied on a sample with certain structural characteristics, which may have limited the examination of the relationship between goal orientation and perceived stress, as well as the moderating role of proactive personality, across different groups (e.g., gender and socioeconomic status). Future research could benefit from expanding the sample size and increasing participant diversity through more refined sampling strategies, thereby enhancing the methodological rigor and generalizability of the findings.

## 5 Conclusion

In summary, this study draws the following conclusions: Among university students, Mastery-approach, Mastery-avoidance, Performance-approach, and Performance-avoidance goals all positively predict Challenge stressors and Hindrance stressors, suggesting that the effects of achievement goal orientation are context-dependent and not universally protective. Proactive personality plays an important moderating role in the relationship between achievement goal orientation and perceived stress. For Challenge stressors, its effect is significant only under Performance-approach goals, attenuating the positive impact on stress, whereas no significant moderation occurs under Mastery-approach, Mastery-avoidance, or Performance-avoidance goals. For Hindrance stressors, Proactive personality generally weakens the positive influence of all achievement goal orientations, consistent with cognitive appraisal theory, indicating that proactive traits help individuals cope more effectively with potentially threatening stressors.

## Data Availability

The raw data supporting the conclusions of this article will be made available by the authors, without undue reservation.
